# virusMED: your travel guide to the virus world

**DOI:** 10.1107/S2052252521011350

**Published:** 2021-11-01

**Authors:** Fasséli Coulibaly

**Affiliations:** a Infection Program, Biomedicine Discovery Institute and Department of Biochemistry and Molecular Biology, Monash University, Clayton, VIC 3800, Australia

**Keywords:** virus hotspots, viral protein structures, epitopes, antiviral drugs, DrugBank, viral metal proteins, virusMED database

## Abstract

As we respond to viral epidemics and accelerate the discovery of new viruses, sifting through vast volumes of structural virology data could rapidly become an impossible task. virusMED is a curated atlas of metal/drug-binding and immunological hotspots in viral protein structures that provides a navigation guide for structure–function analysis and the development of antiviral strategies.

The virus world is wide and vast. And we have just started venturing further in our explorations through metagenomics studies and the characterization of the multitude of variants in viruses relevant to human health.

Fortunately, help is coming to explore the virosphere – another term used to describe the virus world – in the form of a new server called virusMED (*M*etal binding sites, antigenic *E*pitopes and *D*rug binding sites, https://virusmed.biocloud.top) developed by Wladek Minor, Heping Zheng and their collaborators (see Zhang *et al.*, 2021 ▸, in this issue of **IUCrJ** ).

To understand the scale of the task at hand, viruses infecting bacteria, called phages, alone represent the most abundant biological entity on earth, outnumbering cellular organisms by a factor of 10 (Dion *et al.*, 2020 ▸). They have been less studied than their medically relevant counterparts but a flood of data has begun, owing in part to the renewed interest in phage therapy to combat antimicrobial resistance (Gordillo Altamirano & Barr, 2019 ▸) and the proposed role of phages in regulating the population dynamics of bacteria playing an important role in carbon capture (Suttle, 2005 ▸). Sampling of our oceans, soil and even our own gut has already expanded our view of phage diversity and generated masses of viral and virus-like sequences (Paez-Espino *et al.*, 2016 ▸; Camarillo-Guerrero *et al.*, 2021 ▸; Dion *et al.*, 2020 ▸).

The search for viruses that infect eukaryotes is equally raging. For some discoveries, like mimiviruses and related giant viruses (La Scola *et al.*, 2003 ▸), the biological significance is complex but they represent a wealth of new proteins, novel biological processes and almost infinite research questions. For others, the impact is immediately evident such as the characterization of the RNA virome in animals that represent known reservoirs for zoonotic viruses such as flaviviruses, haemorrhagic fever viruses, influenza viruses and coronaviruses.

As a tool for exploration, sequencing is extremely powerful in organizing viruses into families and setting up a robust classification of these organisms in the ever-growing Taxonomy of Viruses (https://talk.ictvonline.org). A complementary approach has focused on the determination and cataloging of three-dimensional structures produced by viruses. Indeed, structure determination of intact viruses has been at the forefront of developments in structural biology since its birth (Harrison, 2015 ▸; Rossmann, 2013 ▸). These structures are made freely available to all through the Protein Data Bank (Johnson & Olson, 2021 ▸), which celebrates 50 years of existence this month, as well as the virus particle explorer database (VIPERdb; http://viperdb.scripps.edu) (Montiel-Garcia *et al.*, 2021 ▸). These invaluable resources are highly curated and constant efforts have been made to improve the quality of their content and make it more accessible to all despite exponential growth (Smart *et al.*, 2018 ▸; Montiel-Garcia *et al.*, 2021 ▸).

While these resources are free, analysis of each viral structure is onerous and requires expertise in several inter-connected fields such as biochemistry, chemistry, molecular virology and immunology. This is where virusMED comes in to save the day. Think of it as your GPS navigation app that will guide you quickly and safely to your destination. Like a navigation app, it taps into tools that are readily available elsewhere: primary databases with sequence information, 3D structures, functional sites, metal and drug binding sites, and epitope repositories. The power of the server is to help researchers combine information from these different sources and make sense of it for the specific goal of understanding and combating viruses [Fig. 1 ▸(*a*)].

Taking SARS CoV-2 as an example, in the space of 21 months (February 2020–October 2021) over 117 000 publications were made available in PubMED and 1555 structures in the PDB. This represents an average of over 180 articles and 2 new structures every day. Organizing, curating and providing visualization tools for this large amount of data is essential to extract the most relevant information in a format that will help generate novel insights [Fig. 1 ▸(*b*)].

virusMED provides an integrated portal to navigate through 7041 structures across 75 viral families. One can browse the database using several pre-set entry points or design specific searches to rapidly gain an overview of where and how metals and small molecules bind to a specific target. Many of these ‘hotspots’ will be important functionally and may represent targets for drug development. With drug repurposing in mind, the results can be filtered to focus only on known drugs found in Drugbank or those that are already FDA approved.

The same portal allows mapping of antigenic sites in viral proteins, providing a database of more than 5000 B- and T-cell epitopes for 329 individual proteins. These can be combined to determine the antigenic landscape of viral proteins, identify variants likely to escape vaccination or reveal targets for potent and broadly neutralizing epitopes.

As the database grows, future developments can be anticipated such as more complex visualization options and an integrated search engine allowing the comparison of new structures with known hotspots. Data on binding sites for other viral proteins and cellular factors accumulate rapidly (Goodacre *et al.*, 2020 ▸; Gordon *et al.*, 2020 ▸) and, while a huge task, would deserve a similar atlas. For now, there is little doubt that many in the structural virology community will adopt this tool to accelerate their research and facilitate the development of antiviral strategies.

## Figures and Tables

**Figure 1:**
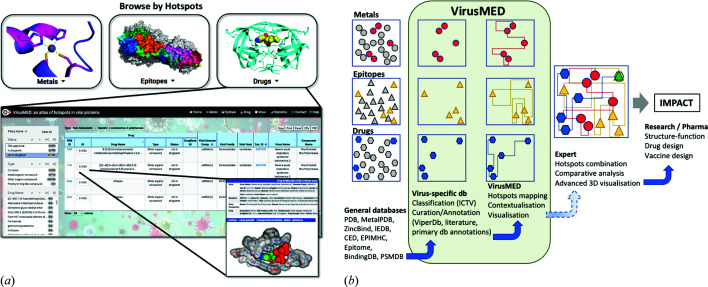
(*a*) virusMED is an atlas of hotspots present in viral proteins that correspond to metal binding sites, epitopes and drugs/small molecules. It is searchable by virus (not shown) and type of hotspot (top panel). Results are presented in a tabular form that can be filtered (middle panel) and mapped onto the 3D structure, providing context and a detailed view of the hotspot. (*b*) Schematic loosely based on a DIKW hierarchy. virusMED provides a navigation tool tailored to molecular virology that consolidates curated data available in various databases. This atlas is likely to facilitate research for viral families with a high volume of data, where expert analysis is time-consuming (coronaviruses, HIV, influenza viruses *etc.*). The dotted lines indicate possible future developments that will further integrate the individual atlases and facilitate comparative analysis (*e.g.* overlapping drug and epitope hotspots shown in green).
